# Survival of metastatic melanoma patients after dendritic cell vaccination correlates with expression of leukocyte phosphatidylethanolamine-binding protein 1/Raf kinase inhibitory protein

**DOI:** 10.18632/oncotarget.18698

**Published:** 2017-06-27

**Authors:** Sonja I. Buschow, Matteo Ramazzotti, Inge M.J. Reinieren-Beeren, Lucie M. Heinzerling, Harm Westdorp, Irene Stefanini, Luca Beltrame, Stanleyson V. Hato, Eva Ellebaek, Stefanie Gross, Van Anh Nguyen, Georg Weinlich, Jiannis Ragoussis, Dilair Baban, Beatrice Schuler-Thurner, Inge M. Svane, Nikolaus Romani, Jonathan M. Austyn, I. Jolanda M. De Vries, Gerold Schuler, Duccio Cavalieri, Carl G. Figdor

**Affiliations:** ^1^ Department of Tumor Immunology, Radboud Institute for Molecular Life Sciences, Radboud University Medical Center, Nijmegen, The Netherlands; ^2^ Department of Gastroenterology and Hepatology, Erasmus University Medical Center (Erasmus MC), Rotterdam, The Netherlands; ^3^ Department of Experimental and Clinical Biomedical Sciences, University of Florence, Florence, Italy; ^4^ Department of Dermatology, University Hospital Erlangen, Friedrich-Alexander-University Erlangen-Nuremberg (FAU), Erlangen, Germany; ^5^ Department of Oncology, Istituto di Ricerche Farmacologiche Mario Negri, Milan, Italy; ^6^ CCIT, Center for Cancer Immune Therapy, Department of Hematology and Department of Oncology, Copenhagen University Hospital, Herlev, Denmark; ^7^ Department of Dermatology, Venereology and Allergology, Medical University of Innsbruck, Innsbruck, Austria; ^8^ Genomics Group, Wellcome Trust Centre for Human Genetics, University of Oxford, Oxford, UK; ^9^ Nuffield Department of Surgical Sciences, University of Oxford, John Radcliffe Hospital, Oxford, UK; ^10^ Department of Biology, University of Florence, Firenze, Italy; ^11^ Current address: McGill University and Genome Quebec Innovation Centre, McGill University, Quebec, Canada

**Keywords:** melanoma, dendritic cell vaccination, immunotherapy, immune suppression, PEBP1

## Abstract

Immunotherapy for metastatic melanoma offers great promise but, to date, only a subset of patients have responded. There is an urgent need to identify ways of allocating patients to the most beneficial therapy, to increase survival and decrease therapy-associated morbidity and costs. Blood-based biomarkers are of particular interest because of their straightforward implementation in routine clinical care. We sought to identify markers for dendritic cell (DC) vaccine-based immunotherapy against metastatic melanoma through gene expression analysis of peripheral blood mononuclear cells. A large-scale microarray analysis of 74 samples from two treatment centers, taken directly after the first round of DC vaccination, was performed. We found that phosphatidylethanolamine binding protein 1 (*PEBP1)*/Raf Kinase inhibitory protein (RKIP) expression can be used to identify a significant proportion of patients who performed poorly after DC vaccination. This result was validated by q-PCR analysis on blood samples from a second cohort of 95 patients treated with DC vaccination in four different centers. We conclude that low *PEBP1* expression correlates with poor overall survival after DC vaccination. Intriguingly, this was only the case for expression of *PEBP1* after, but not prior to, DC vaccination. Moreover, the change in *PEBP1* expression upon vaccination correlated well with survival. Further analyses revealed that *PEBP1* expression positively correlated with genes involved in T cell responses but inversely correlated with genes associated with myeloid cells and aberrant inflammation including *STAT3, NOTCH1*, and *MAPK1*. Concordantly, PEBP1 inversely correlated with the myeloid/lymphoid-ratio and was suppressed in patients suffering from chronic inflammatory disease.

## INTRODUCTION

Metastatic melanoma is one of the most devastating types of cancers in terms of potential life-years lost and affects a growing number of patients each year [1, 2]. Currently, there are different types of immunotherapy that may offer long-term benefit for melanoma patients. These therapies are based on: 1) antagonists of molecules that suppress pre-existing anti-tumor immune responses, so-called immune checkpoint inhibitors such as anti-CTLA-4 (ipilimumab) and anti-PD-1/PD-L1 antibodies (nivolumab/pembrolizumab/atezolizumab); 2) delivery of autologous *ex vivo* expanded tumor infiltrating lymphocytes to boost anti-tumor T cell response; 3) oncolytic viruses injected into metastases to lyse tumor cells and enhance immune responses; and 4) *ex vivo* or *in vivo* targeting of dendritic cell (DCs) to initiate and/or boost tumor antigen-specific immune responses (reviewed in [3, 4]). These new immunotherapies, however, are extremely expensive and/or labor intensive due to high production costs and/or the need for patient-personalized preparation. In addition, although these therapies offer clear benefit for a group of patients, there are also many primary and secondary non-responders [5–7]. This is illustrated by the fact that individual melanoma patients can respond very well to immunotherapy, but those that do represent a limited proportion of the total number who receive this treatment [8–13]. Markers to determine which patients do, or do not, benefit are therefore urgently needed to facilitate treatment decisions. In particular, mechanism of action-based markers, derived from our understanding of why specific treatments are beneficial or not in certain patient populations, have a high potential [7].

So far, a number of prognostic factors for survival in melanoma have been defined, together with several markers associated with or predicting responses to various forms of therapy [14–17]. The success of many immunotherapies is thought to be associated with the composition and organization of the tumor microenvironment, and therefore biomarkers are often sought in this respect [18]. Indeed several studies have identified tumor properties that have correlated the with outcome of immunotherapies, including the expression of cytokines (interferon-γ), chemokines (CCL5, CXCL9, CXCL10), immunoregulatory molecules such as indolamine-2,3-dioxygenase [8, 19] and, more recently, tumor expression of PD-L1 that was reported to be predictive for responses to checkpoint inhibitors in melanoma [20].

Many of the potential biomarkers for immunotherapy against melanoma, reported so far, have been tissue based. This severely limits their clinical applicability because for many patients the primary tumor is not or no longer available, and metastatic tumor tissue material can also be hard to obtain. Furthermore, intra-individual heterogeneity of tissue biomarker expression (i.e. between different metastases and/or with respect to the primary tumor) may limit reliability [21]. These factors can severely limit the predictive effectiveness of a biomarker and, more importantly, may lead to the incorrect exclusion of patients from a potentially effective therapy. Furthermore, tissue biomarkers can only be assessed at one single time point. In contrast, blood-based markers can potentially overcome all these issues. Diagnostic liquid biopsies have already been used in breast cancer, colorectal cancer and in non-small cell lung cancer [22–24]. Moreover, blood samples are being utilized for cell-based cancer-detection in plasma and for tumor mutational analyses [24–26]. A major advantage of obtaining blood samples as a source of biomarkers for therapy prediction is that it is a minimally-invasive intervention which allows the tracking of disease dynamics during the course of treatment.

For dendritic cell DC vaccination specifically, efforts have been made to identify markers associated with patient responses to therapy [27, 28]. In our laboratory, we have developed a delayed type hypersensitivity response (DTH) test to assess the induction of tumor-specific T cells in response to DC vaccination into the skin [29, 30]. This showed that the induction of antigen-specific T cells by DC vaccination was associated with improved survival. However the absence of detection of tumor-reactive T cells did not necessarily indicate a poor response, and therefore additional methods to predict whether a patient will benefit from DC therapy are required. Here we have used microarray analysis of peripheral blood mononuclear cells (PBMCs) from treated patients to identify biomarkers that can be used to monitor and further improve our understanding of the response to DC vaccination. Using this strategy we have identified and validated phosphoethanolamine binding protein 1 (*PEBP1*), also known as Raf-1 kinase inhibitor protein (RKIP), as a biomarker that can be used to predict patient survival after DC vaccination. *PEBP1* has previously been described to modulate several major cancer and inflammatory signaling pathways [31–34]. Here, further in-depth analysis of the genes that are co-expressed with *PEBP1* suggests that its increased expression after vaccination is indicative of the beneficial skewing of the adaptive immune system towards an effective anti-tumor response.

## RESULTS

### Selection of genes with prognostic potential from microarray data of PBMCs from treated patients

To identify biomarkers in the blood of treated patients that may correlate with survival after DC vaccination, we analyzed PBMCs from a total of 74 patients by microarray (MA). These patients had been treated with DC vaccination in two different centers (Discovery cohort; Nijmegen and Erlangen). Only PBMC samples taken after vaccination were at our disposal in sufficient numbers to allow MA-based comparison of good and poor responders. In search of a reasonably-sized set of potential biomarkers that allowed validation by qPCR, we performed several different statistical analyses, including survival analysis, statistical analysis of microarray (SAM) as well as multivariate analysis (PLS-DA; Supplementary Figure 1). For survival analysis, expression data were related to survival time after the start of DC vaccination in a continuous manner. For SAM and PLS-DA, patients were grouped into three survival subgroups [short (<1 year); medium (1-2 years), and long (>2 years); Supplementary Table 1)], to allow us initially to search for gene expression differences between the extremes of this survival spectrum. For SAM, we extracted probes with expression values satisfying two criteria: (i) significant differences between patients from the short and long survival subgroups; and (ii) significant differences between PBMCs and vaccine DCs of the same donor (to increase the chance of finding biomarkers related to a specific cell type and/or immune response). For all three methods, a final filter taking at least a 2-fold change in expression levels between the short and long survival subgroups was applied (Supplementary Figure 1). These three approaches resulted in relatively large lists ranging from 62 to 307 candidates that all may have potential prognostic value. To further reduce the list to a size compatible with qPCR validation, only the candidates that emerged from all three approaches were retained. This final list consisted of 19 genes (Table 1).

**Table 1:** Validation of MA expression data by qPCR

Gene (Symbol)	Pearson correlation MA / qPCR	p-value	Spearman correlation to survival	p-value
***FCGR1B***	**0.68**	**< 0,0001**	**-0.52**	**0.0023**
***ANXA2***	**0.47**	**0.0059**	**-0.46**	**0.0065**
***PEBP1***	**0.46**	**0.0064**	**0.46**	**0.0074**
***IMPA2***	**0.45**	**0.0086**	**-0.34**	**0.0407**
*MNDA*	0.58	0.0006	−0.31	0.055
*SLC4A7*	0.49	0.004	0.29	0.0679
*BST1*	0.45	0.0078	−0.26	0.0898
*MGST1*	0.48	0.0048	−0.24	0.1074
*ZNF467*	0.38	0.0244	0.09	0.3288
*OAS2*	0.42	0.0136	−0.02	0.4637
*UBE2L6*	0.33	0.0428	0.02	0.4681
*ATP1B3*	0.20	0.1505	NA	NA
*FH*	0.16	0.2113	NA	NA
*LILRA5* (primer 1)^a^	0.31	0.0527	NA	NA
*LILRA5* (primer 2)^a^	0.28	0.0779	NA	NA
*TPM3* (probe 1)^b^	−0.16	0.2147	NA	NA
*TPM3* (probe 2)^b^	−0.10	−0.0967	NA	NA
*SRSF6*	0.01	0.4705	NA	NA
*MAT2B*	−0.04	0.4255	NA	NA
*PSMB9*	−0.11	0.2933	NA	NA
*TLR6*	0.14	0.2389	NA	NA

For the total 28 samples from stage IV patients of the Nijmegen discovery cohort we calculated the correlation between the expression levels as measured by microarray analysis (MA) against those obtained by qPCR. For validated genes we also calculated the correlation between qPCR expression and survival time. Only *FCGR1B, ANXA2, PEBP1 and IMPA2* were validated (in bold). ^a^ Two different primers were tested. ^b^Two probes for this gene were put forward by our statistical pipeline. See also Supplementary Figures 2 and 3 for correlation plots. NA: Not analyzed.

### Validation of microarray analysis results by qPCR

Next, we set out to verify MA results using qPCR. For MA analysis we had chosen to use as many datasets as possible in order to find the most robust differentially-expressed genes across treatment centers, and to add power to the statistical analysis. This strategy, however, posed the risk of a center effect since the samples from the two treatment centers were not fully comparable in terms of patient composition and timing of sample collection (Supplementary Materials and Supplementary Tables 1-3). In addition, while stage IV patients comprised the majority, stage III melanoma patients had also been included (Supplementary Tables 1 & 2). Thus, to exclude any confounding effects of these dissimilarities, only Nijmegen-center stage IV patients were used for the initial qPCR validation of the MA result and for the subsequent selection of qPCR validated genes. For 28 patients within this group, we first directly compared the MA data to the qPCR data obtained, to assess which genes behaved most consistently using these very different techniques. Out of 19 genes derived by MA, 11 showed a significant and positive correlation with the qPCR measurement (Supplementary Figure 2 and Table 1).

We then assessed whether the expression of any of the above qPCR-validated genes also correlated with survival. After correction for multiple testing, four genes out of the 11 passed this criterion (Supplementary Figure 3 and Table 1). Only one of these genes, *PEBP1* (RKIP) correlated positively with patient survival; the remaining three, *FCGR1B* (CD64), *ANXA2* (Annexin-2) and *FCGR1B* (Fcγ-receptor 1B, CD64), correlated negatively (Table 1). Hence these four genes could potentially predict clinical outcomes and/or provide mechanistic insights into the mechanisms of patient responses to DC therapy.

### Validation of differential expression of selected genes between patient groups by qPCR

**Figure 1:**
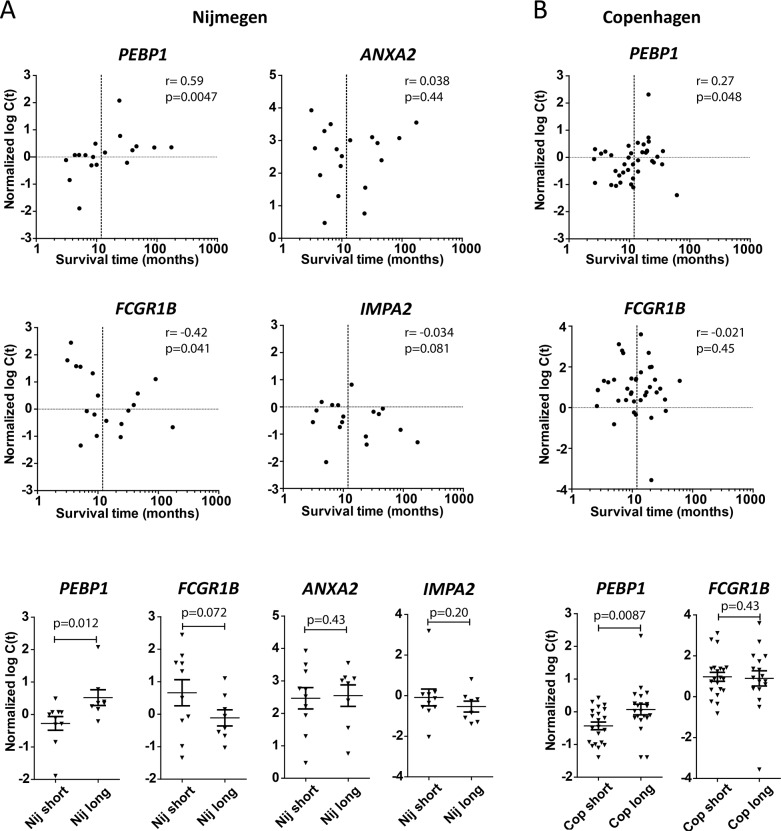
Validation on independent samples Samples from Nijmegen (Nij) that had not been part of the microarray (MA) study **(A)** and samples from a third completely-independent treatment center, Copenhagen (Cop; **B**), were tested to validate the MA-predicted association with patient survival. Upper panels: expression (qPCR) versus survival time. Spearman r and p-value (one-tailed) are given. Lower panels: expression (qPCR) of indicated genes in short (<1 year) versus long (>1 year) surviving patients in the two treatment centers. p-value by t-test (one-tailed).

We wished to further assess and validate the association of the above 4 genes with patient survival after DC vaccination. Therefore we tested their expression on a second novel set of samples derived from Nijmegen patients that had not been used for MA gene selection, and on a completely independent set of samples derived from Copenhagen, a third treatment center exploiting DC vaccination. Interestingly, for *PEBP1* the expected positive correlation with survival time, as well as a significantly lower expression in PBMCs from short surviving patients, was observed in validation samples from both treatment centers (Figure 1). Also, additional samples not used for MA from the Erlangen treatment center showed a similar trend for *PEBP1* despite the fact that the timing of sample collection was very different from the other two treatment centers (Supplementary Figure 4 & Supplementary Materials). Unfortunately, insufficient samples from short survivors were available from Erlangen to draw any firm conclusions. For *FCGR1B*, a trend for negative association with survival was observed, but only in the Nijmegen validation cohort (Figure 1). However, the association of *ANXA2* and *IMPA2* could not be confirmed in any of these samples: they were neither differentially expressed between long and short survivors in the additional Nijmegen samples, nor in the completely independent Copenhagen samples (Figure 1A and data not shown).

### Assessment of the clinical value of PEBP1 as a biomarker

**Figure 2:**
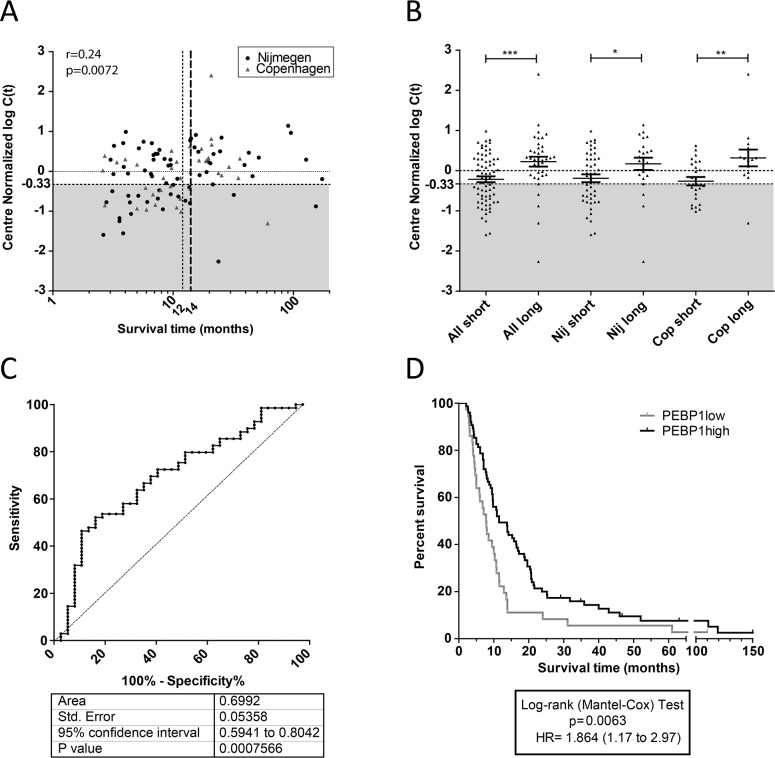
Expression of *PEBP1* is of value for treatment decision **(A)** Spearman correlation of center-normalized expression values for *PEBP1* with survival time after DC vaccination (one-tailed p-value). Grey area: population of patients with low expression of *PEBP1* (<-0.33) that have predominantly a limited survival. Specifically indicated are the original 12-month (dotted line) and the adjusted 14-month (broken line in bold) survival windows. **(B)** Center-normalized expression of *PEBP1* in all evaluated patient samples from Nijmegen and Erlangen (discovery and validation together; one-sides p-values by t-test). **(C)** Reciever operater curve (ROC) of all Nijmegen and Copenhagen samples combined to select short survivors based on low expression of *PEBP1*. **(D)** Kaplan-Meyer curve of patients having a *PEBP1* expression level below or above −0.33 (2 log center normalized C(t)).

Having established that the patients performing poor after DC vaccination expressed lower levels of *PEBP1* in PBMCs acquired after vaccination, irrespective of the treatment center, we set out to quantify the efficacy of *PEBP1* to discriminate short- and long-surviving patient populations. To this end, we first performed a normalization step based on the median value of *PEBP1* measured on all samples from each center and subsequently merged all Nijmegen and Copenhagen data (Figure 2; Supplementary Table 1 for patient numbers). We then correlated the normalized expression data from all patients with survival time. On this merged qPCR dataset, containing all stage IV patient samples from Nijmegen and Copenhagen, we again observed a significant correlation of *PEBP1* expression with survival, confirming the potential of *PEBP1* as a biomarker (Figure 2A). By visualizing the total patient population in one plot, it became apparent that, while most well-responding patients displayed a high expression of *PEBP1*, poorly responding patients could be subdivided into two groups with either low or high *PEBP1* expression. This indicates that while low *PEBP1* expression for the vast majority of patients marks a poor clinical outcome after DC vaccination, high PEBP1 expression does not necessarily indicate a good prognosis. Nevertheless, a low expression of PEBP1 after the first set of vaccinations could identify potential non-responders early-on, and thus enable early change to alternative or additional treatment. To find the optimal cut-off level of *PEBP1* expression and survival length to identify such patients, we generated receiver-operator curves (ROC). The ROCs were based on the predetermined 12-month cut-off (i.e. the short survival group). This ROC performed well [area under the curve (AUC) 0.67+/−0.053]. The correlation plot, however, indicated that a survival of 14 months rather than 12 months best delineated the patients with low *PEBP1* expression and low survival. Indeed, patient selection was further improved when the maximal survival time for the short survival group was extended to 14 months (Figure 2A). This yielded the largest differences between survival groups and a ROC curve with an AUC of 0.69+/−0.053 (Figure 2B and 2C). Of note, similar or better results were obtained for each treatment center separately, yielding AUC of 0.67+/−0.06 and 0.76+/−0.08 for Nijmegen and Copenhagen respectively (Supplementary Figure 5). Also, the Erlangen dataset showed the same trend (AUC 0.64+/−0.14) but the ROC result was not significant, likely because of the low number of short surviving patients in this cohort (7 patients). Based on the ROC curve of all pooled samples we determined that a *PEBP1* expression cut-off of −0.33 (center normalized ^2^log(Ct)) allowed us to identify patients who were surviving less than 14 months after vaccination with a sensitivity of 46% and a specificity of 89%. Using this expression cut-off we were thus able to select a patient population with a significantly poorer clinical outcome after vaccination, that is unlikely to respond to further DC therapy in the applied format and which would be in immediate need of other or additional treatments (Figure 2D).

### PEBP1 expression during the course of vaccination

**Figure 3:**
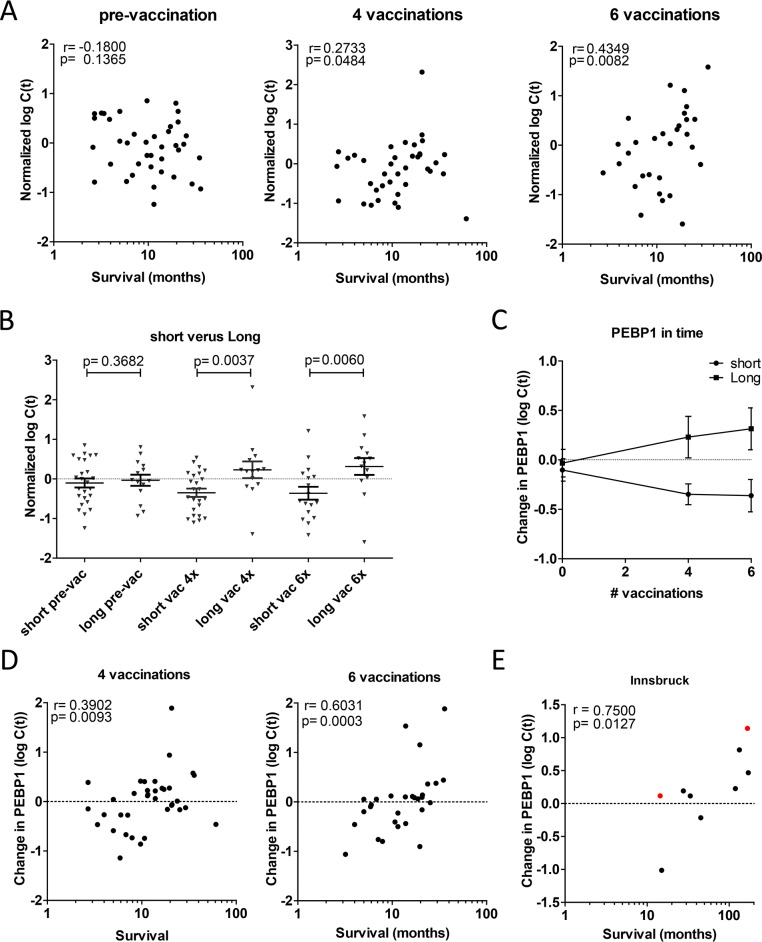
Change in PEBP1 expression correlates with survival after vaccination **(A-D)** Expression of PEBP1 was assessed in Copenhagen patients prior to or after 4 or 6 rounds of vaccination. (A) Spearman correlation of PEBP1 at the 3 time-points (p-value one-tailed). Respectively n=39, n=38 and n=30 pre-, after 4 and after 6 vaccinations. (B) Expression of PEBP1 at each time-point in short (<14 months) or long survivors (> 14 months). Ns= non-significant * p<0.05 ** p<0.01 by t-test (one-tailed). (C) Course of PEBP1 expression during successive rounds of vaccination in short and long survivors (Mean +/− SEM). (D) Spearman correlation of survival time with the change in PEBP1 expression with respect to the start of vaccination (p-value one-tailed). E Change in PEBP1 expression in relation to survival in patient samples from Innsbruck (n=7 stage IV in black; n=2 stage III in red).

We then investigated whether *PEBP1* expression levels could be informative also before vaccination, and therefore studied the expression of *PEBP1* over time. For this we used Copenhagen samples for which PBMCs were taken a few days before vaccination, after 4 vaccinations and after 6 vaccinations. Intriguingly, there were no differences in the expression levels of short and long surviving patients in PBMCs before vaccination, indicating *PEBP1* expression correlates with clinical outcome after vaccination but cannot *a priori* predict whether a patient will respond or not (Figure 3A and 3B). The difference in *PEBP1* expression was further increased after two additional vaccinations (Figure 3B and 3C). Notably, we found that the change *of PEBP1* in response to vaccination correlated better with survival time than the expression level of *PEBP1* itself (Figure 3D). Interestingly, an effect of vaccination was seen both in the short- and long-surviving patients: in short-surviving patients PEBP1 levels decreased during the course of vaccination, whereas it increased in long survivors (Figure 3C). A similar trend was also present in a few longitudinal samples available from a fourth, independent treatment center in Innsbruck (Figure 3E). Together these results suggest that the change in *PEBP1* expression (change) is indicative of patient responses to DC vaccination using overall survival as an endpoint, and as such may provide a mechanistic insight into what determines a successful treatment response.

### PEBP1 may indicate skewing of the immune response

**Figure 4:**
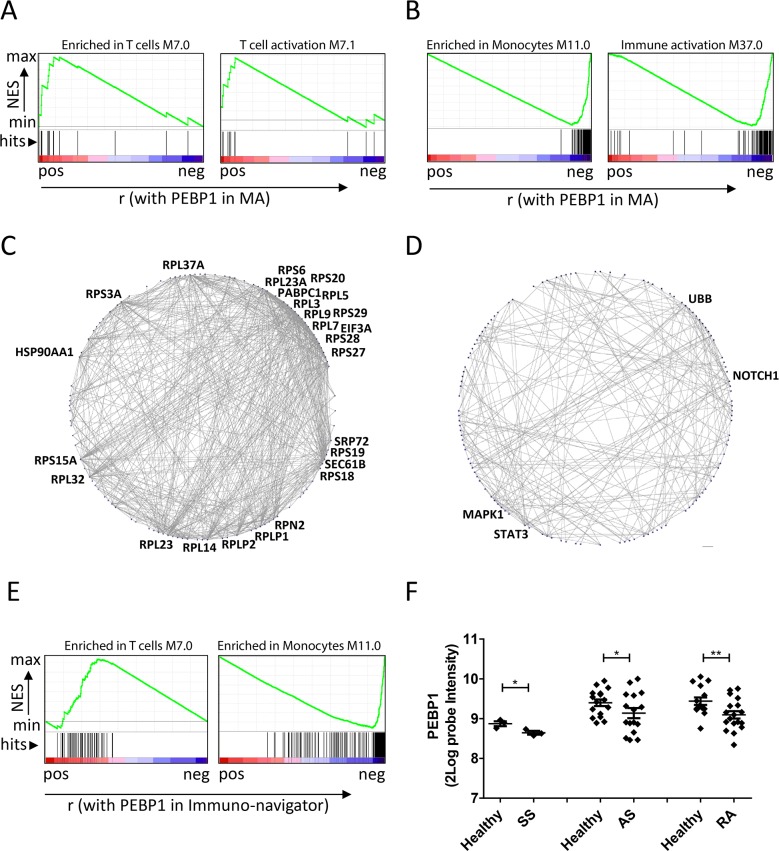
*PEBP1* expression reflects metabolic and inflammatory state **(A** and **B)** Examples of BTMs that were found enriched by GSEA in the genes correlating to PEBP1 in the microarray dataset. Genes positively correlated to *PEBP1 (A)* were enriched in BTMs related to T cell (activation), whereas those negatively correlating with *PEBP1* were enriched in BTMs related to monocytes and general inflammation (A). **(C** and **D)** Protein-protein Interactions (PPI) present in the STRING database for the top 500 genes positively (C) or negatively correlating to PEBP1 (D). Shown are genes for which the STRING database reported an interaction with a high confidence score (0.7) and more than one connection. Gene symbols are only displayed for genes with at least 20 (left graph) or 10 connections (right graph). **(E)** Enrichment of genes from the T cell and monocyte module within a list of genes ranked on correlation with PEBP1 in the Immuno-Navigator database. F) Expression of PEBP1 in blood or PBMC samples of healthy donors versus patients suffering from Ankylosing spondylitis (AS; GDS5231), Schnitzler syndrome (SS; GSE70019) and rheumatoid arthritis (RA; GDS3794) as retrieved using GEO2R.

To obtain further insights into how *PEBP1* expression may reflect the response to DC vaccination, we interrogated the original MA data to identify genes that that were positively or negatively correlated with *PEBP1* to understand more about the molecular context in which this protein may act. First, we analyzed genes ranked on their correlation with PEBP1, for the enrichment of previously described blood transcriptional modules (BTMs) using Gene set enrichment analysis (GSEA) [35–37]. Interestingly, BTMs indicated that *PEBP1* expression correlated well with genes associated with an adaptive immune response (e.g. T cells and their activation) as well as the regulation of transcription (Table 2; Figure 4). However, the negatively-correlated genes were enriched for those relating to myeloid cells (e.g. monocytes, neutrophils, and immature DCs) and inflammation.

**Table 2:** GSEA for blood transcriptional modules on correlation of genes with *PEBP1* expression

Correlation to PEBP1	Blood transcriptional module	# genes	NES	FDR q-val
Positive	ENRICHED IN T CELLS (I) (M7.0)	15	2.11	0.000
	T CELL ACTIVATION (I) (M7.1)	11	1.80	0.011
	NUCLEAR PORE COMPLEX (M106.0)	10	1.58	0.036
	REGULATION OF TRANSCRIPTION, TRANSCRIPTION FACTORS (M213)	11	1.54	0.035
Negative	ENRICHED IN MONOCYTES (II) (M11.0)	95	−3.18	0.000
	CELL CYCLE AND TRANSCRIPTION (M4.0)	85	−2.95	0.000
	IMMUNE ACTIVATION - GENERIC CLUSTER (M37.0)	93	−2.80	0.000
	MONOCYTE SURFACE SIGNATURE (S4)	48	−2.75	0.000
	TLR AND INFLAMMATORY SIGNALING (M16)	29	−2.60	0.000
	ENRICHED IN MONOCYTES (IV) (M118.0)	22	−2.48	0.000
	MYELOID CELL ENRICHED RECEPTORS AND TRANSPORTERS (M4.3)	20	−2.40	0.000
	ENRICHED IN NEUTROPHILS (I) (M37.1)	18	−2.31	0.000
	ENRICHED IN MYELOID CELLS AND MONOCYTES (M81)	10	−2.03	0.000
	RESTING DENDRITIC CELL SURFACE SIGNATURE (S10)	14	−2.03	0.000
	REGULATION OF ANTIGEN PRESENTATION AND IMMUNE RESPONSE (M5.0)	31	−2.00	0.000
	ENRICHED IN ANTIGEN PRESENTATION (II) (M95.0)	11	−1.83	0.001
	ENRICHED IN ACTIVATED DENDRITIC CELLS (II) (M165)	11	−1.65	0.006

From the original microarray analysis, genes were ranked based on their correlation with PEBP1 expression and used as input for GSEA using blood transcriptional modules (BTMs) as gene sets. NES: normalized enrichment factor FDR: false discovery rate.

In the search for more mechanistic insights, we subjected the genes that were positively- or negatively-correlated with PEBP1 to protein-protein-interaction and gene ontology (GO) analyses. We found that genes correlating with PEBP1 expression mapped to extremely well-connected, mostly ribosomal, proteins, suggesting high protein synthesis (ability) in these patient samples. Negatively-correlating genes were clustered around the signaling proteins STAT3, NOTCH1, MAPK1 and UBB (Figure 4). Moreover, these genes mapped to GO terms relating to antigen processing and presentation and the response to stress (Supplementary Table 5), and in addition to the innate immune response as previously also indicated by GSEA. Together these results indicate that low *PEBP1* may represent a state of systemic inflammation and stress accompanied by low protein biosynthesis. This systemic state may hamper an effective response to DC vaccination.

To investigate whether these functional implications of PEBP1 expression may stretch beyond DC vaccination and/or metastatic melanoma, we interrogated a large co-expression database of immune cells (immune-navigator) and found that also in this database PEBP1 expression correlated with adaptive immune cell responses and anti-correlated with monocytes and generic immune activation (Figure 4 & Supplementary Table 6). Moreover, further analysis of publicly available blood or PBMC expression data comparing healthy subjects to those suffering from chronic inflammatory diseases (e,g Ankylosing spondylitis, Schnitzler syndrome and rheumatoid arthritis) revealed that low PEBP1 expression may be a more frequent event associated with chronic inflammation than previously anticipated (Figure 4).

**Figure 5:**
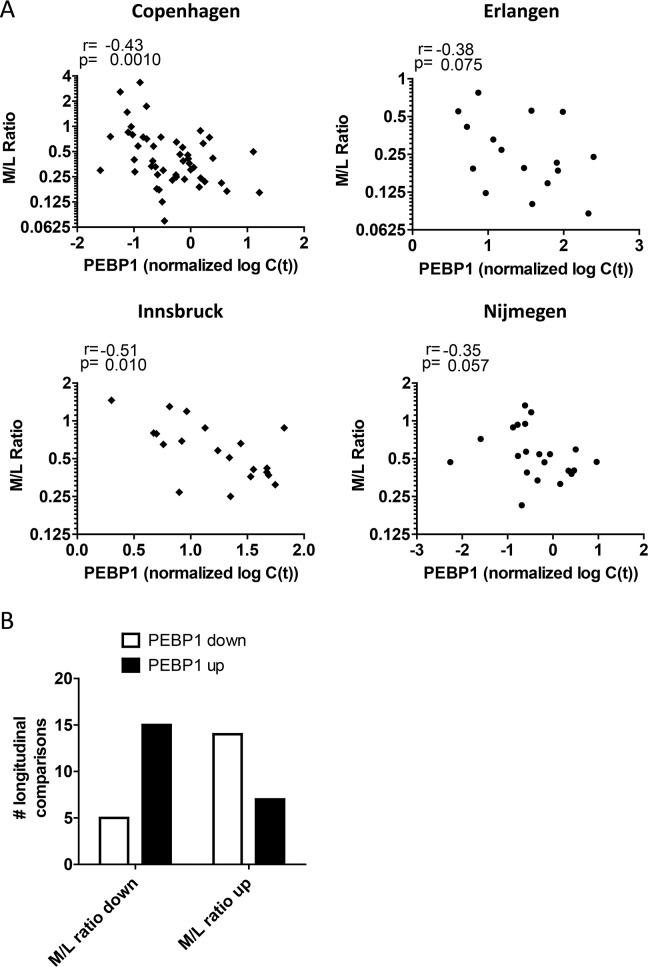
Blood PEBP1 expression anti-correlates with the myeloid/lymphoid balance **(A)** Spearman rank correlation of PEBP1 expression with the myeloid/lymphocyte (M/L) balance determined by flow cytometry at the time of sample collection for all four treatment centers. **(B)** Quantification of the direction of change of PEBP1 with respect to the M/L balance in sequential longitudinal samples from Copenhagen (see also figure 3).

These data suggest that low *PEBP1* expression after DC vaccination may reflect the ineffective development of a productive, adaptive immune response and instead may be associated with myeloid cells that can negatively regulate immune responses in a state of aberrant/chronic inflammation. Recently, several studies have reported the deleterious effect of myeloid-derived suppressor cells (MDSCs) in melanoma and in response to DC vaccination [15, 17, 38, 39]. Indeed, we found that biobanked PBMCs from DC-vaccinated patients demonstrated a trend for the inverse relation between *PEBP1* expression levels and MDSC frequencies in the Nijmegen samples. However, this relationship could not be confirmed for samples of the other centers, possibly due to the non-optimal conditions induced by sample freezing and differences in the MDSC identification strategy (Supplementary Figure 6). In search for an alternative way to further substantiate the notion that high PEBP1 expression may associate with high lymphocyte/adaptive immune responses but low myeloid/innate immune response we re-analyzed all available flow cytometry data of our patients acquired at the same moment of mRNA sample collection to quantify myeloid and lymphoid cell fractions. In agreement with the GSEA result PEBP1 anti-correlated (or showed this trend) with the myeloid/lymphoid balance in all centers tested (Figure 5). This is further supported by the observation that, a change in PEBP1 expression as a result of vaccination was significantly more often associated with a change of the myeloid/lymphocyte balance in the opposite direction (p<0,01 by Fisher exact test; Figure 5).

Together, these data demonstrate that in patients doing poorly after DC vaccination, low PEBP1 levels are indicative of a distorted myeloid/lymphocyte balance and that this may correlate with skewing of the induced immune response towards chronic inflammation.

## DISCUSSION

We here describe *PEBP1* as a novel gene whose low expression on blood leukocytes is associated with poor survival in metastatic melanoma patients receiving DC therapy. The association of *PEBP1* with survival provides important clues towards the mechanisms that determine the success of the DC vaccine-driven immune response. Furthermore, *PEBP1* expression on white blood cells may have clinical value in selecting patients who may not benefit from further DC treatment in its current form and who might be better treated with other forms of immune or combination therapy (for example in combination with immune checkpoint blockade or chemotherapy).

We tested and validated the association of PEBP1 with survival on independent samples from different treatment centers. Our data indicate that low *PEBP1* levels after DC vaccination, but not before, are associated with a poor survival. Interestingly, we found that in patients who responded poorly, *PEBP1* levels decreased even further, whereas patients who did well demonstrated a rise in *PEBP1* levels upon vaccination. Based on the molecular context in which we find *PEBP1* to be expressed, we postulate that a decrease in *PEBP1* may be indicative of a skewing of the (DC-vaccine-triggered) immune response towards chronic inflammation/myeloid immune suppression rather than towards an effective anti-tumor response. Such a scenario would be in line with both the requirement for a robust adaptive response to combat the tumor, and the reported high prevalence of inflammation and/or tumor-induced MDSCs in patients with a poor prognosis [15–17, 38, 39]. Indeed, in the Nijmegen cohort, *PEBP1* levels were negatively correlated with monocytic MDSC numbers. These experiments that aimed to determine the relationship between PEBP1 and MDSC are however by no means conclusive, and were hampered by great variability in MDSCs numbers between centers. The use of frozen material and the non-standardized MDSC identification methods used limited these analyses [40]. Going forward these analyses should be included in future studies. Intriguingly, and in agreement with the reported function and ontogeny of genes correlating and anti-correlating with PEBP1, we found that PEBP1 expression levels in patient samples from all centers was inversely correlated with the myeloid/lymphoid balance in PBMCs at the time of sample collection.

It remains to be determined whether *PEBP1* is directly driving the skewing of T cell-mediated immunity towards robust anti-tumor responses, or is merely a bystander gene reflecting the state of the immune system. *PEBP1*, also known as RKIP, is a mostly-cytoplasmic protein that is widely expressed and has diverse functions affecting several important signaling cascades including NFκB, MAPK/ERK and GSK3β signaling, to regulate cell proliferation, migration, and activation (reviewed by [34, 41]). In cancer, *PEBP1* expression seems mostly favorable to survival and, for melanoma, an inverse correlation between *PEBP1* expression and metastases has been reported [31, 32]. Although we discovered a similar favorable association between high *PEBP1* expression and survival, our finding is on white blood cells, not on tumor cells. Our results are therefore unlikely to be explained by an intrinsic effect of *PEBP1* on tumor cells or their metastatic counterparts. Instead it is more likely that PEBP1 acts on the white blood cells themselves. This notion is supported by the inverse correlation of PEBP1 expression with the myeloid/lymphocyte balance. Intriguingly, the genes inversely correlated with PEBP1 included, and were centered around *STAT3*, *NOTCH1*, and *MAPK1* (ERK2) (by PPI). Possibly the discriminative power of PEBP1 demonstrated here may relate to the signaling pathways associated with these molecules. *PEBP1* expression has been reported to dampen the inflammatory response, acting on both ERK/MAPK and NFκB signaling to reduce pro-inflammatory cytokine release [33, 34, 42, 43]. High *PEBP1* expression has also recently been shown to attenuate *STAT3* signaling, which in turn has been shown to be a key factor for the suppressive effect of MDSCs [44–46]. MDSCs are postulated to dampen the immune response to tumors, including melanoma, and are associated with poor survival [38, 39, 47, 48]. The presence of chronic inflammation in cancer patients has been held responsible for the induction of MDSC via growth factors and cytokines (e.g. G- or GM-CSF, IL-6, TGFβ, PGE2) acting via ERK/mTOR, STAT, NFκB and SMAD signaling pathways [49, 50]. Considering the inverse correlation between PEBP1, STAT3 and ERK expression identified here, it seems quite possible that *PEBP1* acts on these signaling pathways to prevent/dampen excessive systemic inflammation that may hamper an effective adaptive immune response following DC vaccination. This hypothesis is supported by our finding that PEBP1 levels are also diminished in patient suffering from other diseases associated with chronic inflammation.

Besides regulating inflammation in general, the action of *PEBP1* may be more sophisticated. In CD8+ T cells *PEBP1* expression is associated with enhanced IFN-γ signaling downstream of the TCR [51]. *PEBP1* may thus have dual functions in inhibiting deleterious systemic inflammation that drives immunosuppression, whilst at the same time promoting adaptive responses. In line with this view, we find in our gene expression data an inverse correlation of *PEBP1* with genes connected to innate and inflammatory responses, while genes associated with T cell responses positively correlate with *PEBP1* expression. Furthermore, we observed that PEBP1 is also highly correlated with the expression of ribosomal subunits suggesting PEBP1 expression to be associated with high protein biosynthesis capacity. This may facilitate an effective adaptive immune response which is highly dependent on the production of large amounts of inflammatory mediators (e.g. cytokines, antibodies) and immune cell expansion. Further research on the expression and function of *PEBP1* in different immune cells is required to shed further light on how exactly *PEBP1* relates to the skewing of immune responses following DC vaccination.

Our study once more indicates that the presence of excessive/chronic inflammation and /or a lack of “fitness” of the immune system may be a severe obstacle to the success of DC vaccination. Suppressive and exhausted immune cells may hamper the effectiveness of treatment modalities that are aimed at inducing or boosting adaptive immune responses. Together, the data presented here and from other studies, suggest that the presence of chronic inflammation, MDSCs and now also low *PEBP1* expression may all represent contraindications for further DC vaccination in its current form. More studies are required to obtain detailed mechanistic insight into the interaction of these factors. Recent literature suggests that lifting this state of aberrant inflammation and concordant suppression of immune responses in cancer patients, by e.g. interfering with STAT signaling or by certain regiments of chemotherapy could increase the effectiveness of various types of immunotherapy [49, 52, 53]. As such *PEBP1* could represent a facile and effective blood-based biomarker to monitor whether the fitness of the immune system is adequate for successful immunotherapy.

## MATERIALS AND METHODS

### Patients

Clinical data and biological samples from melanoma patients undergoing DCs vaccination were obtained from the University of Erlangen, Erlangen, Germany (see Table 1; www.clinicaltrials.gov NCT00053391 and [54, 55], from the Radboud University Nijmegen Medical Centre, Nijmegen, The Netherlands (NCT00243594; NCT00243529; NCT0228541 and [56–58]), from Copenhagen University Hospital, Herlev, Denmark (NCT00197912 and [59, 60]) and from the Medical University of Innsbruck (patient treatment not part of a clinical trial but as “compassionate use” according to Austrian national regulations; the use of cell samples from these patients for this study was approved by the local ethical committee (reference number AN2016-0130/363/4.18)). All studies were approved by the local regulatory committees, and written informed consent was obtained from all patients.

For our studies, we used PBMCs and DC vaccines stored in biobanks collected during various previously-performed clinical trials. Vaccination strategy in brief: all patients were vaccinated with monocyte-derived dendritic cells generated *ex vivo* from autologous monocytes by culture with GM-CSF and IL-4 and subsequently matured with a cytokine cocktail consisting of IL-1 beta, IL-6, TNF-alpha and PGE_2_ (see references [54–60] for center specific details on DC culture and the origin of used reagents; Erlangen and Innsbruck patients were in part not previously reported and center-specific details on DC generation for these patients are given in Supplementary Methods). In summary, vaccine DCs were either loaded with tumor antigen-derived HLA-specific/matched peptides from P53, Survivin, Telomerase, MAGEA1, MAGEA3, Tyrosinase, MAGEA10, NY-ESO-1, MelanA, and/or gp100, electroporated with mRNA coding for tumor antigens or loaded with tumor cell lysate ([30, 54, 59–61]); Supplementary Table 3). Furthermore, some Erlangen patients were vaccinated with moDCs exposed to the maturation cocktail plus soluble trimeric CD40L (Gross et al., manuscript submitted), and some Copenhagen patients received metronomic doses of cyclophosphamide co-therapy (Supplementary Table 2, Supplementary Materials and [60]). Nijmegen and Copenhagen PBMC were always acquired after an initial cycle of 3 (Nijmegen) or 4 (Copenhagen) vaccinations, at the moment of the delayed type hypersensitivity skin test which is routinely performed 1 week after the last vaccination of this cycle. Additional patient samples from Copenhagen were collected just prior to vaccination or after a total 6 of vaccinations. For the Erlangen cohort, the timing of blood sample collection varied and was on average after 6 vaccinations (range 1-23 vaccinations) that were taken on average 39 days after the last vaccination (range 6-110 days). Innsbruck cohort sample collection was at multiple time points during ongoing vaccination as indicated in Supplementary Figure 6. The Erlangen and Innsbruck cohorts contained many fewer short-surviving patients which might be explained by the fact that vaccination continued after trial end (Supplementary Table 2).

### Microarray data generation

RNA was extracted from biobanked PBMCs and moDCs using the Ambion RNA mirvana™ extraction kit. RNA quality and abundance were determined using an Agilent 2100 Bioanalyzer and Nanodrop ND-1000 spectrophotometer, respectively. 500ng of total RNAs were reverse transcribed to synthesize first- and second- strand complementary DNA (cDNA), purified and *in vitro* transcribed to synthesize biotin-labeled complementary RNA (cRNA) using the Illumina TotalPrep-96 RNA Amplification Kit (# 4393543 Ambion, Inc., Austin, TX). A total of 750ng of biotin-labeled cRNA was then hybridized to Illumina HumanHT-12 V3.0 expression BeadChips at 55C for 18 h. The hybridized BeadChip was washed and stained with streptavidin-Cy3 according to the manufacturers protocols using Illumina whole-genome gene expression direct hybridization assay (#11286340 Illumina, San Diego, California, USA). GenomeStudio Data Analysis Software used to visualize and analyse images generated. This software provides data in standard file formats that can be readily processed with most commercial and/or public accessible gene expression analysis software programs to identify significantly differentially expressed genes with pathways and networks involved. Raw data from BeadStudio were exported as text files and processed using the Bioconductor R package Lumi [62]. Quality controls were performed, and samples with incorrect parameters were discarded. The background was adjusted by subtracting an offset estimated based on the quantile of the control probes; then normalization was performed using log2 transformations. All further statistical analyses, unless otherwise specified, was performed on probe expression values. Raw data as well as normalized data for all the samples (74 PBMC samples and 68 matched mature moDCs samples) we used in this work have been deposited in the ArrayExpress repository with the ID E-MTAB-5201.

### Microarray data statistical analysis

#### Survival analysis by SAM

The significance analysis of microarrays (SAM) as implemented in the Bioconductor R package Rsam [63] includes a procedure for estimating survival associated probes given a vector of survival months and the censoring status of the PBMC donor. We used a total of 74 PBMC samples to draw the survival analysis, keeping the 90^th^ percentile FDR = 0 to exclude the presence of false positive survival associated probes. The resulting probes were then associated with a fold change by dividing their mean expression values in long survivors by their mean expression value in short survivors. A fold-change cut-off of 2 was then applied to restrict the number of probes.

#### Partial least square discriminant analysis/multivariate analysis

Gene expression was also investigated through the multivariate methods implemented in the R package mixOmics [64]. In particular, we used a partial least square analysis (PLS) coupled to a discriminant analysis (DA) to accommodate a two or three-class categorization of the samples. The categories we used were the above mentioned ranked survival classes of long, medium and short survivors. Fold changes were calculated as described for the survival analysis by SAM and filtered at a 2 fold-change cut-off.

#### Two pass / two-class SAM

For this analysis two consecutive two-class SAMs were drawn: in the first pass, we searched for a “vaccination signature” by testing for differential expression in PBMCs obtained from donors after vaccination and the mature DCs included in the vaccine. Since each patient received autologous *in-vitro* matured DCs, we performed a paired PBMC vs matured DC two-class analysis. Only significant probes were analyzed in the second pass, consisting in an unpaired two-class (long vs short survivors) SAM. Also, in this case, we selected probes at 90^th^ percentile FDR = 0 to statistically exclude the detection of false positive results. A 2 fold change cut-off was used for further selection.

### Identification of valid housekeeping reference genes for qPCR normalization from microarray data

Established housekeeping genes for qPCR normalization of PBMCs samples are not consistently reported in literature. Genes that are routinely used as reference genes such as GAPDH do not perform well on whole blood or PBMC samples [65, 66]. We therefore employed our microarray dataset to extract housekeeping genes to be used as a reference for qPCR. We first scanned the expression values of genes available in the microarray platform across all PBMC samples, searching for invariance (standard deviation < 5 %) in all genes with a reasonable deviation from background expression level, to avoid the selection of non-expressed genes. Best-performing genes were then further screened for absence of long/short survivors trends by analyzing both the significance of T-tests and Mann-Whitney tests, the slopes associated to a linear regression of long versus short survivor samples and maximal overlap between the long and short survivor samples. Via this method we isolated PBGD, EEF1A1 and OXSR1 genes as most stably expressed genes. After measuring by qPCR the expression levels of these MA-derived three genes plus the frequently reported GAPDH gene in samples already used in microarrays, we used the procedure described by Vandesopele and coworkers that relies on pairwise variations across samples to rank HKs genes according to their stability and perform a progressive, stepwise elimination [67]. Following this selection, two reference genes were validated, namely OXSR1 and PBGD, and used in combination as reference genes for further qPCR analysis. All the statistics were calculated using the R software.

### RNA isolation and quantitative PCR on patient PBMCs

PBMCs were obtained from each of the Center's Biobanks. RNA was isolated using the RNeasy mini kit (Qiagen, array samples) or Trizol (Life Technologies, additional qPCR samples) following manufacturers protocol. RNA quantity was determined on NanoDrop 2000c (Thermo Scientific) and RNA quality was determined via agarose gel electrophoresis. 2 ug of RNA was DNAse I treated to remove residual genomic DNA and reverse transcribed into cDNA by M-MLV reverse transcriptase (Life Technologies) to obtain 25ul of cDNA. cDNA was diluted 25x in nuclease free water. For each reaction, 4ul diluted cDNA, 300nM primers, 10ul SYBR Green (Roche) and water were added to a final volume of 20ul. Each sample was amplified using a CFX96 sequence detection system (BioRad). The following qPCR cycling conditions were used: 50°C/2min, 95°C/10min, 40cycles of 95°C/15s; 60°C/1min, melt analysis 60°C - 95°C with increment 0.5°C/5s. The gene-specific oligonucleotide primers used to determine the expression of the genes of interest are listed in Supplementary Table 4. To increase the chance of consistency qPCR primers were based on the MA probes with highest differential expression. PCR products were monitored by measuring the increase in fluorescence caused by binding of SYBR Green. Quantitative PCR data were analyzed using CFX96 manager and relative expression of the gene of interest was determined using the cycle threshold method (Livak KJ, 2001) with PBGD and OXSR1 as reference genes. Statistical analysis of normalized qPCR data was performed using graph pad prism software using the statistical tests indicated in the figure legends.

### Gene set enrichment analysis of genes correlating with PEBP1 expression, protein-protein interaction, and gene ontology analysis

Using the full microarray dataset, the Pearson correlation and p-value of all probes in relation to PEBP1 expression on the array was determined in the R programming environment. Genes were ranked based on their log-transformed Pearson correlation coefficient, and this ranked list was used as input for Gene Set Enrichment Analysis (GSEA) against the blood transcriptional modules described by LI at al. [35–37]. Alternatively a ranked list of the correlation of human genes with PEBP1 in the immune-navigator database ((http://sysimm.ifrec.osaka-u.ac.jp/immuno-navigator/) was used as input. For GSEA standard operating setting was applied taking into account only the highest ranking gene sets with a minimum 10 genes.

To build PPI networks the top 500 of genes, correlating or anti-correlating with PEBP1 expression were loaded into STRING database (http://string-db.org) to find any direct (physical) or indirect (functional) associations [68]. Using this database we simultaneously mapped genes on gene-ontologies. PPIs with a high confidence score were loaded in Biolayout express^3D^ for visualization (http://www.biolayout.org/) [69].

## SUPPLEMENTARY FIGURES AND TABLES











## Abbreviations

DTH: delayed type hypersensitivity response
DC: dendritic cell
MA: microarray
MDSCs: myeloid-derived suppressor cells
PBMCs: peripheral blood mononuclear cells
PEBP1: phosphatidylethanolamine binding protein 1
RKIP: Raf kinase inhibitory protein
ROC: receiver-operator curves
SAM: statistical analysis of microarray
