# Supplementary material for: Survival of metastatic melanoma patients after dendritic cell vaccination correlates with expression of leukocyte phosphatidylethanolamine-binding protein 1/Raf kinase inhibitory protein
Source: Oncotarget. 2017 Jun 27;8(40):67439–56. doi: 10.18632/oncotarget.18698 (PMC5620184; doi:10.18632/oncotarget.18698)
Supplement: Supplementary file 1 [file oncotarget-08-67439-s001.pdf]

Survival of metastatic melanoma patients after dendritic cell vaccination correlates with expression of leukocyte phosphatidylethanolamine-binding protein 1/Raf kinase inhibitory protein

SUPPLEMENTARY MATERIALS

Details of not-previously reported clinical trials from Erlangen and Innsbruck

In Erlangen, PBMCs were isolated from blood using a density gradient (Lymphoprep® ; Axis Shield, Oslo). After washing, cells were frozen in human serum albumin (Baxter, Heidelberg), DMSO (Sigma Aldrich, Irvine) and glucose-solution (Glucosteril®, Fresenius, Bad Homburg) and stored in the gas phase of liquid nitrogen. MoDCs were generated as described previously [1, 2]. Monocytes were isolated from initial apheresis and differentiated using GM-CSF (Leukine®) and IL-4 (Cellgenix, Freiburg) for 6 days to generate immature dendritic cells. A maturation cocktail containing IL-1β (Cellgenix, Freiburg), IL-6 (Cellgenix, Freiburg), TNF-α (Beromun®, Boehringer Ingelheim, Ingelheim), and PGE2 (ProstinE2®, Pfizer, Vienna) was added to obtain mature DC on day 7. For peptide loading peptides (Clinalfa-Bachem, Laeufelfingen) were added for 4 hours. Loaded DC were washed and frozen similar to PBMC.

Cocktail-matured moDCs were loaded with 4 HLA-A1 and/or 4 HLA-A2.1-restricted tumor peptides, including cancer testis and melanocyte differentiation antigens: HLA-A1 (MAGE1 = EADPTGHSY, MAGE3 = EVDPIGHLY, Tyrosinase 243-251 = KSDICTDEY, Tyrosinase 146-156 = SSDYVIPIGTY), HLA-A2.1 (MAGE10 = GLYDGMEHL, gp100 ana = IMDQVPFSV, MelanA ana = ELAGIGILTV, NY-ESO1 ana = SLLMWITQV) whereby each class I peptide was loaded onto a separate batch of 12 million cryopreserved DCs to avoid competition for the same class I HLA molecules (www.clinicaltrials.gov, NCT00053391, [3]). In addition, 6 HLA class II tumor peptides (Tyrosinase.DR4 = SYLQDSVPDSFQD, MAGE3.DR13 = LLKYRAREPVTKAE, MAGE3.DP4 = KKLLTQHFVQENYLEY, gp100.DR4 = WNRQLYPEWTEAQRLD, MAGE3.DR11 = TSYVKVLHHMVKISG and NY-ESO1.DP4 = SLLMWITQCFLPVF) were used, loaded on DC independent of HLA type. For part of the patients half of the matured moDC were pulsed with soluble trimeric CD40L and the two DC vaccines (with and without trimeric CD40L) were administered separately into the deep dermis of each thigh (i.e. right and left) or, when lymphadenectomy had been performed, at the upper arms). For the second cohort of patients no CD40L was added to the moDC, but the moDC to be loaded with class I HLA-A peptides were exposed to KLH protein (2µg/ml) to provide unspecific help for CTL induction. DC to be loaded with HLA class II peptides were not loaded with KLH.

Methods for the generation and the quality control of DCs in Innsbruck were essentially the same as in Erlangen. Trimeric CD40 ligand was not used in the Innsbruck cohort.

Enumeration of myeloid derived suppressor cells and the myeloid lymphoid ratio

For quantification of the frequency of monocytic MDSCs of Nijmegen patients, PBMCs from DC vaccinated patients (after vaccination) were thawed and split in two, one fraction for RNA isolation and qPCR quantification of PEBP1 expression (main manuscript) and one part for immunostaining with FVD eFluor® 780 (aBioscience), CD14-FITC (Miltenyi) and HLA-DR PerCP (biolegend) to identify MDSCs as the FVD-CD14+HLA-DRlow population. Monocytic and granulocytic MDSCs of Copenhagen patients were enumerated in a previous publication [4]. MDSCs from the Erlangen cohort were stained using a lineage-/ dump channel with PerCP-conjugated antibodies for CD3, CD19, CD56 (all from Biolegend) and 7AAD as dead cell marker and in addition CD11b-BV605, CD11c-BV510, CD14-BF711, CD16-BUV395, CD33-APC, CD80-AlexaFluor700, CD83-FITC, CD124-PE, CD274-PE-Cy7 and HLA-DR- APC-Cy7 (all from BD) and identified as Viability dye/Lin-CD14+HLA-DRlow for the monocytic MDSCs and as Viability Dye/Lin-CD33+CD11b+CD14-HLA-DRlow for the granulocytic MDSCs.

To quantify the myeloid/ lymphoid balance any available flow cytometry data acquired of a patient at the time of mRNA sample collection was re-analyzed and the relative number of myeloid and lymphoid cells were determined based in forward and side scatter as described in [5]. For Innsbruck samples datafiles were no longer accessible and previously determined percentages of CD14+ and CD3+CD19+CD56+ populations were used to calculate the percentage of myeloid and lymphoid cells respectively.

REFERENCES

1. Thurner B, Roder C, Dieckmann D, Heuer M, Kruse M, Glaser A, Keikavoussi P, Kampgen E, Bender A, Schuler G. Generation of large numbers of fully mature and stable dendritic cells from leukapheresis products for clinical application. J Immunol Methods. 1999; 223:1-15.
2. Berger TG, Feuerstein B, Strasser E, Hirsch U, Schreiner D, Schuler G, Schuler-Thurner B. Large-scale generation of mature monocyte-derived dendritic cells for clinical application in cell factories. J Immunol Methods. 2002; 268:131-140.
3. Gross S, Erdmann M, Haendle I, Voland S, Berger T, Schultz, E, Strasser E, Dankerl P, Janka R, Schliep S, Heinzerling, L, Sotlar K, Coulie P, et al Twelve-year survival and immune correlates in dendritic cell-vaccinated melanoma patients. JCI Insight. 2017; 2: pii: 91438.
4. Ellebaek E, Engell-Noerregaard L, Iversen TZ, Froesig TM, Munir S, Hadrup SR, Andersen MH, Svane IM. Metastatic melanoma patients treated with dendritic cell vaccination, Interleukin-2 and metronomic cyclophosphamide: results from a phase II trial. Cancer Immunol Immunother. 2012; 61:1791-1804.
5. Welters MJ, van der Sluis TC, van Meir H, Loof NM, van Ham VJ, van Duikeren S, Santegoets SJ, Arens R, de Kam ML, Cohen AF, van Poelgeest MI, Kenter GG, Kroep JR, et al. Vaccination during myeloid cell depletion by cancer chemotherapy fosters robust T cell responses. Science Transl Med. 2016; 8:334ra352.

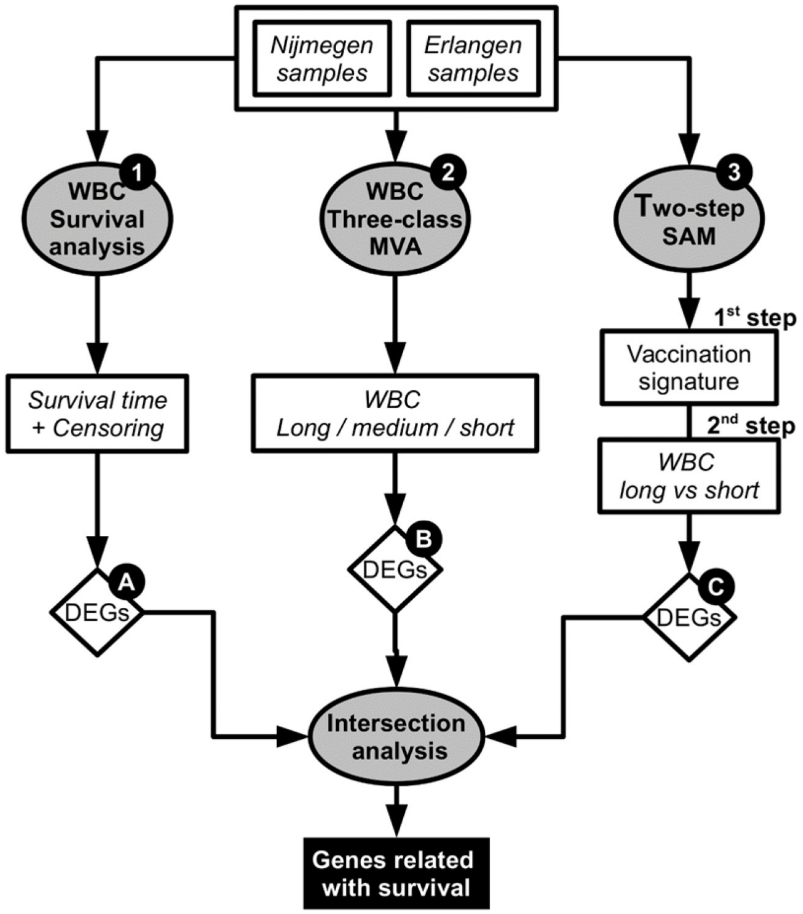

Supplementary Figure 1: Methodology used to derive genes associated with patient survival from microarray data. Microarray data of White blood cells (WBC) from patients after receiving DC vaccination were analysed using three different statistical methods 1) classical survival analysis 2) Multi-variate analysis (MVA) and statistical analysis of microarrays (SAM) (see methods for details). Differentially expressed genes (DEG) identified with all three methods were used for further qPCR validation.

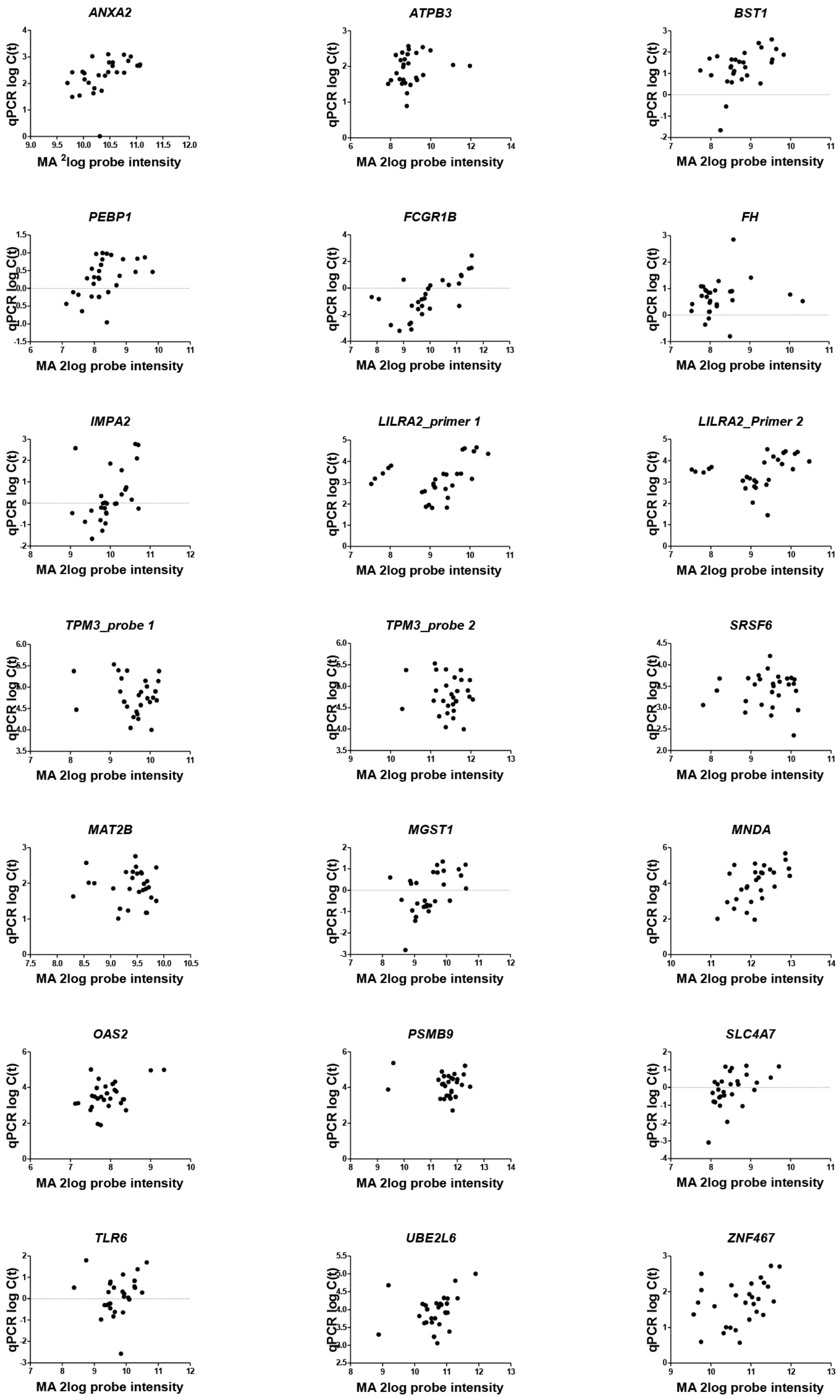

Supplementary Figure 2: Validation of MA expression level by qPCR Of a total 28 samples from stage IV patients of the Nijmegen discovery cohort we compared the microarray expression levels to qPCR expression levels and plotted the results obtained by the two methods against each other.

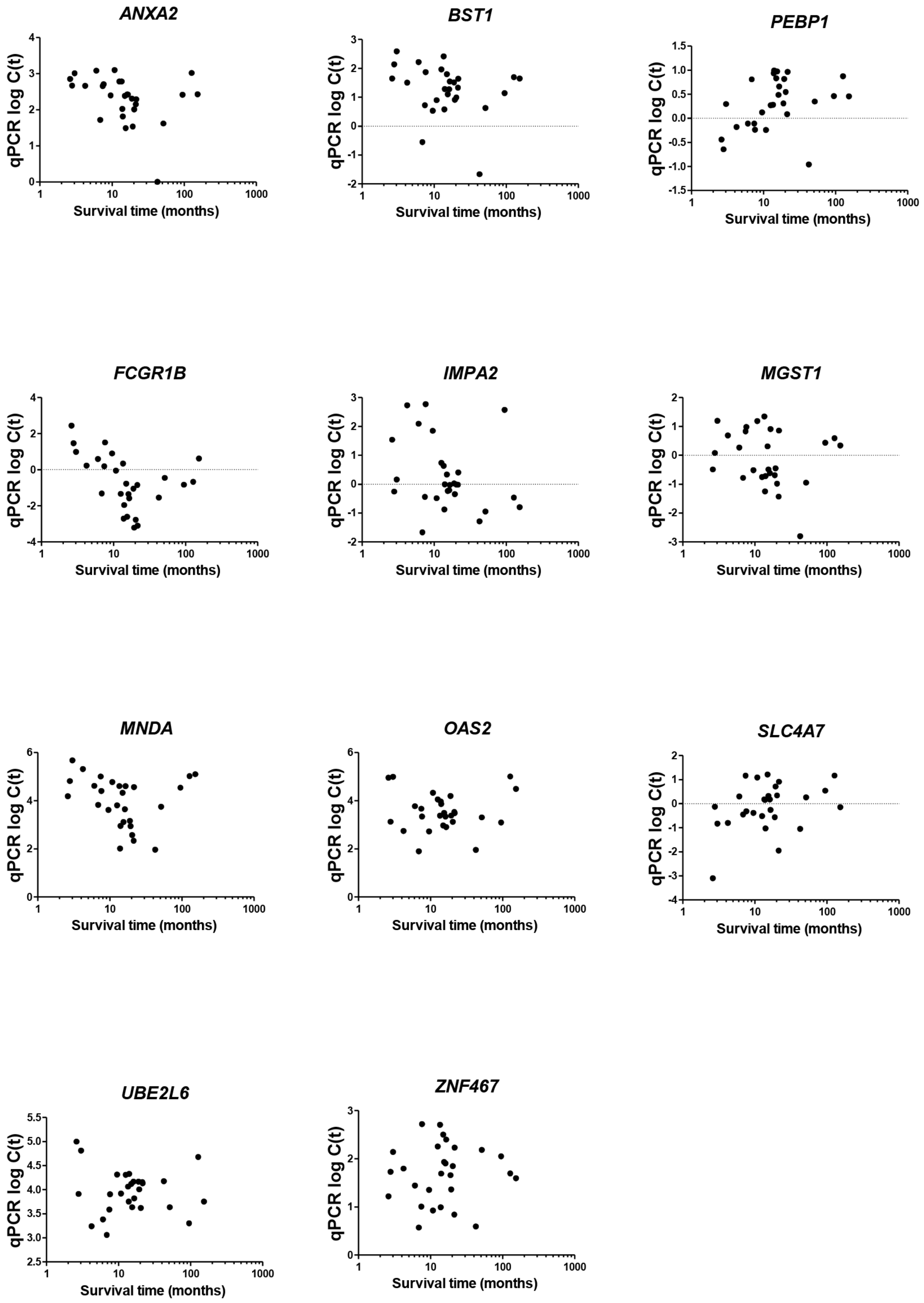

Supplementary Figure 3: Validation of correlation to survival time by qPCR For the total 28 samples from stage IV patients of the Nijmegen discovery cohort we plotted the expression levels measured by qPCR against the survival time.

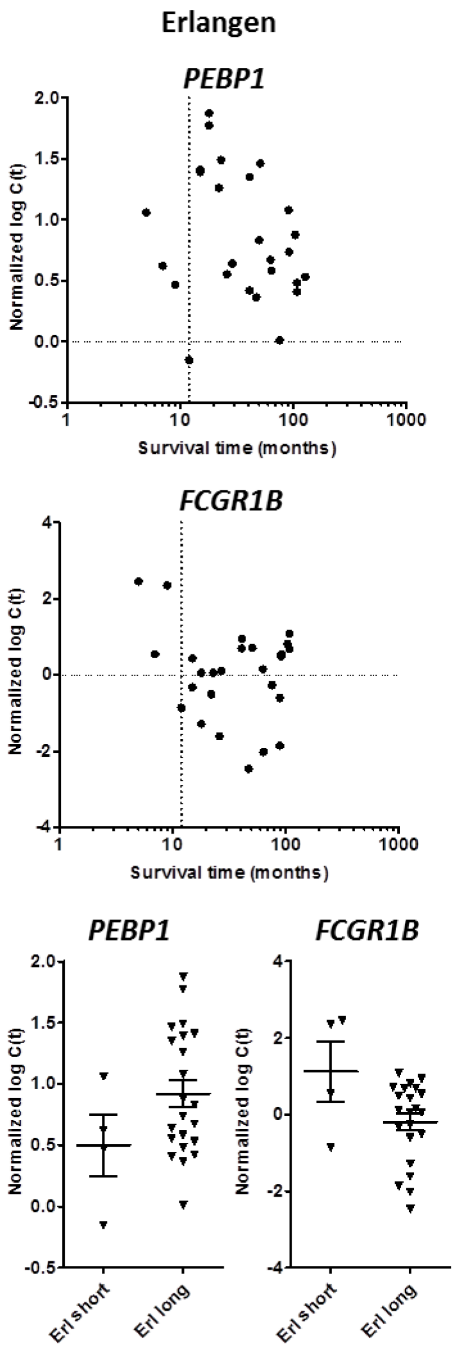

Supplementary Figure 4: Validation on independent Erlangen samples. Samples from Erlangen (Erl) that had not been part of the microarray (MA) study were tested to validate the association of high *PEBP1* and low *FCGR1B* expression with longer patient survival. **(A)** Expression (qPCR) versus survival time. **(B)** Expression (qPCR) of *PEBP1* and *FCGR1B* in short (<1 year) and long (>1 year) surviving patients in the two treatment centers.

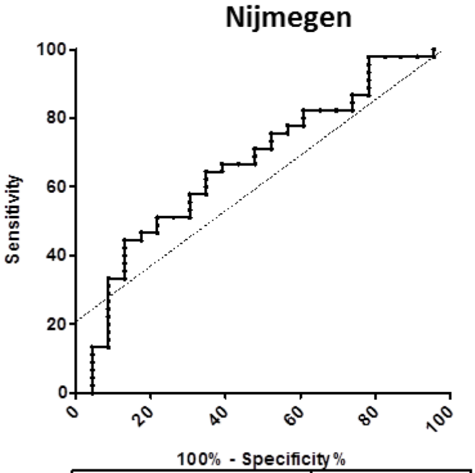

Area	0.6715
Std. Error	0.06922
95% confidence interval	0.5358 to 0.8072
P value	0.02144

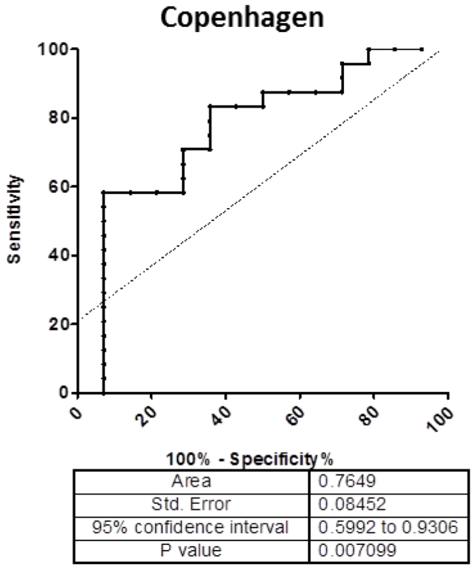

Area	0.7649
Std. Error	0.08452
95% confidence interval	0.5992 to 0.9306
P value	0.007099

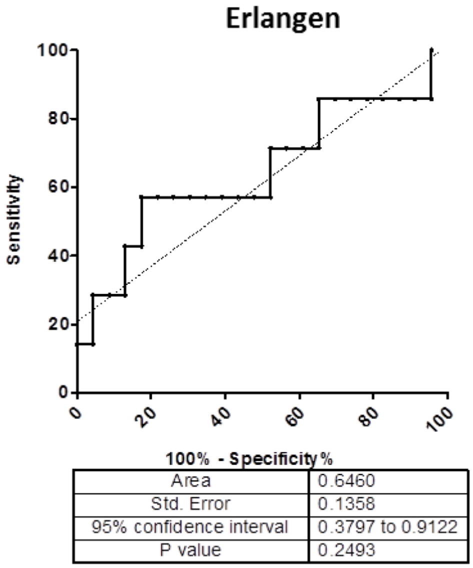

Area	0.6460
Std. Error	0.1358
95% confidence interval	0.3797 to 0.9122
P value	0.2493

Supplementary Figure 5: *PEBP1* based ROC curves for the individual treatment centers. Receiver Operator Curves (ROC) describing the selection of short surviving patients (<14 months) based on *PEBP1* expression for all stage IV patients from each individual treatment center.

Supplementary Table 1: Summary of PBMC samples studied

See Supplementary File 1

Supplementary Table 2: Summary of patient characteristics

Centre	DC antigen loading			Route of administration					# vaccines		Remarks
	Peptide	Lysate	RNA	i.n	i.v and/ or i.d	i.d.	i.d. or i.n.	i.v.	Median	Range	
Copenhagen (n=41)	21	20	0			20	21		6	4 - 19	Part of patients received metronomic doses of cyclophosphamide and Celecoxib in addition to DC therapy (n=20)
Erlangen (n=49)	49	0	0	2	2	45(44*)			15	4-41	Part of patients received moDCs pulsed with CD40L during maturation (n=40)
Innsbruck (n=9	9					9			24	16-57	All patients received either dacarbazine (DTIC) and/or fotemustine chemotherapy co-treatment at conventional doses
Nijmegen (n=70)	49	0	21	43	26	1			3	3 - 11	A few patients received anti-CD25 therapy 4-8 days after vaccination (n=5)

* subcutanous but nearly i.d. positioned injection

Supplementary Table 3: Summary of monocyte derived DC vaccines administrated

Stage	Study part	Method	Location of use in manuscript	Centre	PBMC samples			
					Short <1 year	Long >1 year (>2 years)	Total/ centre	Total/ study part
Stage III/IV	Discovery	MA	Tables 1 & 2, Supplementary Figures 2-4	Erlangen	4	19 (18)	23	74
				Nijmegen	29	22(8)	51	
Stage IV		MA & qPCR (all genes)	Tables 1 & 2, Supplementary Figures 2, 3, 6	Nijmegen	10	18(5)	28	
		MA & qPCR (PEBP1 only)	Figure 2, Supplementary Figure 6	Erlangen	3	1 (1)	4	
				Nijmegen	19	1(1)	20	
	Validation	qPCR	Figures 1-3, Supplementary Figures 5 & 6	Copenhagen	24	17(5)	41	95
			Supplementary Figures 4-6	Erlangen	4	22 (6)	26	
			Figure 3	Innsbruck	0	9	9	
			Figures 1-3, Supplementary Figures 5 & 6	Nijmegen	11	8(6)	19	

Supplementary Table 4: Primer sets used for qPCR

See Supplementary File 2

Supplementary Table 5: Gene ontology analysis on genes correlating positively or negatively with *PEBP1* expression in the microarray analysis.

See Supplementary File 3

Supplementary Table 6: GSEA for blood transcriptional modules on correlation of genes with *PEBP1* expression in the Immune-navigator database .

See Supplementary File 4
