# Supplementary material for: Survival of metastatic melanoma patients after dendritic cell vaccination correlates with expression of leukocyte phosphatidylethanolamine-binding protein 1/Raf kinase inhibitory protein
Source: Oncotarget. 2017 Jun 27;8(40):67439–56. doi: 10.18632/oncotarget.18698 (PMC5620184; doi:10.18632/oncotarget.18698)
Supplement: Supplementary file 2 [file oncotarget-08-67439-s002.docx]

**Supplementary Table 1: Summary of PBMC samples studied**

| **Centre** | **Age (years)** | | **Sex** | | | **OS (months)** | | | **Pre-treatment therapies** | | **Stage** | | | | | | | |
| --- | --- | --- | --- | --- | --- | --- | --- | --- | --- | --- | --- | --- | --- | --- | --- | --- | --- | --- |
|  | median | range | M | F | median | | range |  | | M1a | | M1b | M1c | Mx^a^ | IIIc | IIIb | IIIa | III |
| Copenhagen (n=41) | 59 | 23-76 | 29 | 12 | 11 | | 3-61 | systemic treatment naive n=6, IL-2 and/or IFNa (n=30) chemotherapy (n=9) and/or other kinds of therapy | | 4 | | 9 | 28 |  |  |  |  |  |
| Erlangen (n=49) | 51 | 21-75 | 25 | 24 | 58 | | 6-141 | systemic treatment naive n=15, chemotherapy n=15, IFN-a and/or IL-2 n=21 or other kinds of therapy | | 9 | | 6 | 23 | 3 | 3 | 2 | 2 | 1 |
| Innsbruck (n=9) | 62 | 45-77 | 7 | 2 | 14 | | 14-171 | Systemic treatment naive n=0, radiation n=4, chemotherapy n=8, IFNa and/or IL-2 n=7 or other kinds of therapy | | 4 | |  | 3 |  |  |  |  | 2 |
| Nijmegen (n=70) | 57 | 21- 77 | 48 | 22 | 10 | | 3-173 | systemic treatment naive n= 14, radiation n=26 chemotherapy n=29 and/or other kinds of therapy | | 9 | | 20 | 39 | 0 | 2 |  |  |  |

*^a^Mx = distant metastasis, localization unknown*
