# Supplementary material for: Survival of metastatic melanoma patients after dendritic cell vaccination correlates with expression of leukocyte phosphatidylethanolamine-binding protein 1/Raf kinase inhibitory protein
Source: Oncotarget. 2017 Jun 27;8(40):67439–56. doi: 10.18632/oncotarget.18698 (PMC5620184; doi:10.18632/oncotarget.18698)
Supplement: Supplementary file 3 [file oncotarget-08-67439-s003.docx]

**Supplementary Table 4: Primer sets used for qPCR**

| **Gene** | **Primer name** | **Sequence (5' -3')** | **Protein** |
| --- | --- | --- | --- |
| LILRA5_1 | LILRA5_fwd_1 | GCTCAGATGCTATGGCT | leukocyte immunoglobulin-like receptor, subfamily A (with TM domain), member 5 (LILRA5), transcript variant 2 |
|  | LILRA5_rev_1 | TCTCTACTGCGTAATCCTGAAGGTG |  |
| PSMB9 | PSMB9_fwd_1 | GTGGTGAACCGAGTGTT | proteasome (prosome, macropain) subunit, beta type, 9 (large multifunctional peptidase 2) (PSMB9) |
|  | PSMB9_rev_1 | GGTTCCTCCAGTTCTATCCCATG |  |
| MNDA | MNDA_fwd_1 | GTGTCCCAAGAGCAGAG | myeloid cell nuclear differentiation antigen (MNDA) |
|  | MNDA_rev_1 | TGCTTTCAGTACCACCACTGTCA |  |
| OAS2 | OAS2_fwd_1 | AAGCCCTACGAAGAATGT | 2'-5'-oligoadenylate synthetase 2, 69/71kDa (OAS2), transcript variant 1 |
|  | OAS2_rev_1 | TTGGCTTCTCTTCTGATCCTGG |  |
| FCGR1B | CD64_fwd_1 | TCTGCTCCTTTGGGTT | Fc fragment of IgG, high affinity Ia, receptor (CD64) (FCGR1A) |
|  | CD64_rev_1 | CTGTGCCATTGAGAAACCACTG |  |
| MAT2B | MAT2B_fwd_1 | GGGTTGGAGGAGGTG | methionine adenosyltransferase II, beta (MAT2B), transcript variant 1 |
|  | MAT2B_rev_1 | GGCACCAGTAACCAGAACCCT |  |
| ANXA2 | ANXA2_fwd_1 | CCAGGTTATCTTGTAGCATAG | annexin A2 (ANXA2), transcript variant 2 |
|  | ANXA2_rev_1 | GTTCAAAGCATCCCGCTCAG |  |
| SLC4A7 | SLC4A7_fwd_2 | GCAAATTCCAGTCAAGG | solute carrier family 4, sodium bicarbonate cotransporter, member 7 (SLC4A7) |
|  | SLC4A7_rev_3 | CCGGTAGTCACACATAAACA |  |
| TPM3 | TPM3_fwd_1 | CAGGCAGATGATGCAG | tropomyosin 3 (TPM3), transcript variant 5 |
|  | TPM3_rev_2 | AAGGCCCGGTTTTCAATAA |  |
| TLR6 | TLR1_fwd_2 | TAACAAAGGCATATTGGG | toll-like receptor 6 (TLR6) |
|  | TLR1_rev_2 | GGAACGTGGATGAGACCGTTT |  |
| DUSP6 | DUSP6_fwd_2 | CTGCTCAAGAAGCTCAAG | dual specificity phosphatase 6 (DUSP6), transcript variant 1 |
|  | DUSP6_rev_1 | CACTGGGAAGGAAGGCTGG |  |
| IMPA2 | IMPA2_fwd_4 | ACGGCACCTGCAATTTTGTG | inositol(myo)-1(or 4)-monophosphatase 2 (IMPA2) |
|  | IMPA2_rev_4 | GCTCTTGTCGAACAGCAAATCC |  |
| MGST1 | MGST1_fwd_2 | CAGATGACAGAGTAGAACGTG | microsomal glutathione S-transferase 1 (MGST1), transcript variant 1a |
|  | MGST1_rev_2 | ACTCAAAGCTCTATTTGGCTGGG |  |
| ATP1B3 | ATP1B3_fwd_4 | AACCCGACCACCGGAGAAT | ATPase, Na+/K+ transporting, beta 3 polypeptide (ATP1B3) |
|  | ATP1B3_rev_4 | TGAGAGTCTGAAGCATAACCCA |  |
| BST1 | BST1_fwd_2 | CACCCATCCTGACTGTG | bone marrow stromal cell antigen 1 (BST1) |
|  | BST1_rev_1 | CACAGAACACGAATGATGACTGC |  |
| PEBP1 | PEBP1_fwd_5 | CCTCTCCGATTATGTGGGCTC | phosphatidylethanolamine binding protein 1 (PEBP1) |
|  | PEBP1_rev_5 | CCTGTCCTGCTCGTAAACCA |  |
| SRSF6 | SRSF6_fwd_2 | GAAGCAGATCCAGGTCTC | serine/arginine-rich splicing factor 6 (SRSF6), transcript variant 1 |
|  | SRSF6_rev_2 | CTTCGAGAATGTGAATGGGAGC |  |
| LILRA5_2 | LILRA5_fwd_3 | GACTGAGGAAGGAGACC | leukocyte immunoglobulin-like receptor, subfamily A (with TM domain), member 5 (LILRA5), transcript variant 2 |
|  | LILRA5_rev_3 | GACGGACTGAGGTTATCAGCT |  |
| UBE2L6 | UBE2L6_fwd_2 | GAGTGGTGAAGGAGCTG | ubiquitin-conjugating enzyme E2L 6 (UBE2L6), transcript variant 1 |
|  | UBE2L6_rev_1 | GTCGGGTAGGAGGAGAGCGT |  |
| FH | FH_fwd2 | GCCGCTGAAGTAAACCAGGAT | Fumarate Hydratase (FH) |
|  | FH_rev3 | AACATGATCGTTGGGATGCAC |  |
| ZNF467 | ZNF46_fwd_2 | CTTCTAGGGAAGAGAGAGCA | Zinc finger protein 467 (ZNF467) |
|  | ZNF46_rev_2 | GCTGGGAAGTAACGATAGA |  |
| EEF1A1 | EEF1A1_fwd4 | TGTCGTCATTGGACACGTAGA | Eukaryotic translation elongation factor 1 alpha 1 |
|  | EEF1A1_rev3 | ACGCTCAGCTTTCAGTTTATCC |  |
| OXSR1 | OXSR1_fwd3 | GTGGCAATCAAACGGATAAACC | Oxidative-stress responsive 1 |
|  | OXSR1_rev3 | TGATGGCATTGACTCATGGCT |  |
