# Supplementary material for: Survival of metastatic melanoma patients after dendritic cell vaccination correlates with expression of leukocyte phosphatidylethanolamine-binding protein 1/Raf kinase inhibitory protein
Source: Oncotarget. 2017 Jun 27;8(40):67439–56. doi: 10.18632/oncotarget.18698 (PMC5620184; doi:10.18632/oncotarget.18698)
Supplement: Supplementary file 4 [file oncotarget-08-67439-s004.docx]

**Supplementary Table 5: Gene ontology analysis on genes correlating positively or negatively with *PEBP1* expression in the microarray analysis.**

|  | **ID** | | **Description** | | **# genes** | | **FDR** | |  |
| --- | --- | --- | --- | --- | --- | --- | --- | --- | --- |
| Positive | GO.0016071 | | mRNA metabolic process | | 57 | | 3.72E-20 | |  |
|  | GO.0044260 | | cellular macromolecule metabolic process | | 212 | | 3.78E-16 | |  |
|  | GO.0006139 | | nucleobase-containing compound metabolic process | | 161 | | 5.39E-15 | |  |
|  | GO.0006396 | | RNA processing | | 55 | | 5.39E-15 | |  |
|  | GO.0044238 | | primary metabolic process | | 241 | | 2.23E-14 | |  |
|  | GO.0006725 | | cellular aromatic compound metabolic process | | 162 | | 4.70E-14 | |  |
|  | GO.0044237 | | cellular metabolic process | | 237 | | 4.70E-14 | |  |
|  | GO.0046483 | | heterocycle metabolic process | | 162 | | 4.70E-14 | |  |
|  | GO.0090304 | | nucleic acid metabolic process | | 147 | | 4.70E-14 | |  |
|  | GO.0016070 | | RNA metabolic process | | 135 | | 4.99E-14 | |  |
|  | GO.0010467 | | gene expression | | 146 | | 6.25E-14 | |  |
|  | GO.0043170 | | macromolecule metabolic process | | 216 | | 6.35E-14 | |  |
|  | GO.1901360 | | organic cyclic compound metabolic process | | 166 | | 8.77E-14 | |  |
|  | GO.0016482 | | cytoplasmic transport | | 52 | | 1.55E-13 | |  |
|  | GO.0008152 | | metabolic process | | 256 | | 2.63E-13 | |  |
|  | GO.0006614 | | SRP-dependent cotranslational protein targeting to membrane | | 22 | | 3.55E-13 | |  |
|  | GO.0071704 | | organic substance metabolic process | | 240 | | 3.85E-13 | |  |
|  | GO.0044403 | | symbiosis, encompassing mutualism through parasitism | | 53 | | 1.74E-12 | |  |
|  | GO.0034641 | | cellular nitrogen compound metabolic process | | 168 | | 1.77E-12 | |  |
|  | GO.0016032 | | viral process | | 50 | | 1.95E-12 | |  |
|  | GO.0044265 | | cellular macromolecule catabolic process | | 52 | | 2.32E-12 | |  |
|  | GO.0006807 | | nitrogen compound metabolic process | | 174 | | 4.35E-12 | |  |
|  | GO.1902582 | | single-organism intracellular transport | | 62 | | 3.32E-11 | |  |
|  | GO.0044267 | | cellular protein metabolic process | | 122 | | 5.13E-11 | |  |
|  | GO.0019058 | | viral life cycle | | 28 | | 5.47E-11 | |  |
|  | **ID** | | **Description** | | **# genes** | | **FDR** | |  |
| Negative | | GO.0055114 | | oxidation-reduction process | | 50 | | 3.73E-05 | |
|  |  | GO.0006950 | | response to stress | | 113 | | 6.48E-05 | |
|  |  | GO.0009987 | | cellular process | | 289 | | 2.41E-04 | |
|  |  | GO.0044281 | | small molecule metabolic process | | 78 | | 2.41E-04 | |
|  |  | GO.0051641 | | cellular localization | | 77 | | 2.41E-04 | |
|  |  | GO.1902582 | | single-organism intracellular transport | | 51 | | 2.41E-04 | |
|  |  | GO.0007166 | | cell surface receptor signaling pathway | | 75 | | 2.80E-04 | |
|  |  | GO.0045087 | | innate immune response | | 44 | | 2.80E-04 | |
|  |  | GO.0048002 | | antigen processing and presentation of peptide antigen | | 17 | | 2.80E-04 | |
|  |  | GO.0019882 | | antigen processing and presentation | | 18 | | 4.18E-04 | |
|  |  | GO.0034613 | | cellular protein localization | | 49 | | 4.18E-04 | |
|  |  | GO.0046907 | | intracellular transport | | 54 | | 4.18E-04 | |
|  |  | GO.0033036 | | macromolecule localization | | 73 | | 4.54E-04 | |
|  |  | GO.0045088 | | regulation of innate immune response | | 24 | | 5.41E-04 | |
|  |  | GO.0002478 | | antigen processing and presentation of exogenous peptide antigen | | 15 | | 7.16E-04 | |
|  |  | GO.0006839 | | mitochondrial transport | | 15 | | 7.39E-04 | |
|  |  | GO.0008104 | | protein localization | | 63 | | 7.39E-04 | |
|  |  | GO.0044711 | | single-organism biosynthetic process | | 53 | | 7.39E-04 | |
|  |  | GO.0016043 | | cellular component organization | | 134 | | 1.07E-03 | |
|  |  | GO.0044763 | | single-organism cellular process | | 241 | | 1.23E-03 | |
|  |  | GO.0002376 | | immune system process | | 69 | | 1.83E-03 | |
|  |  | GO.0006952 | | defense response | | 54 | | 1.83E-03 | |
|  |  | GO.0051649 | | establishment of localization in cell | | 63 | | 1.87E-03 | |
|  |  | GO.0009605 | | response to external stimulus | | 66 | | 1.88E-03 | |

Shown is the top 25 scoring terms of each GO analysis.
