# Supplementary material for: Survival of metastatic melanoma patients after dendritic cell vaccination correlates with expression of leukocyte phosphatidylethanolamine-binding protein 1/Raf kinase inhibitory protein
Source: Oncotarget. 2017 Jun 27;8(40):67439–56. doi: 10.18632/oncotarget.18698 (PMC5620184; doi:10.18632/oncotarget.18698)
Supplement: Supplementary file 5 [file oncotarget-08-67439-s005.docx]

**Supplementary Table 6: GSEA for blood transcriptional modules on correlation of genes with *PEBP1* expression in the Immune-navigator database.**

|  | **Name** | **Size** | **NES** | **FDR q-val** |
| --- | --- | --- | --- | --- |
| Positive | CELL CYCLE (I) (M4.1) | 142 | 2.52 | 0.0E+00 |
|  | NUCLEAR PORE COMPLEX (M106.0) | 17 | 2.34 | 0.0E+00 |
|  | RESPIRATORY ELECTRON TRANSPORT CHAIN (MITOCHONDRION) (M238) | 17 | 2.28 | 0.0E+00 |
|  | MISMATCH REPAIR (I) (M22.0) | 27 | 2.27 | 0.0E+00 |
|  | CELL CYCLE (III) (M103) | 51 | 2.23 | 0.0E+00 |
|  | MITOTIC CELL CYCLE - DNA REPLICATION (M4.4) | 28 | 2.19 | 0.0E+00 |
|  | T CELL ACTIVATION (I) (M7.1) | 50 | 2.19 | 0.0E+00 |
|  | MITOTIC CELL DIVISION (M6) | 30 | 2.17 | 0.0E+00 |
|  | PLK1 SIGNALING EVENTS (M4.2) | 33 | 2.16 | 0.0E+00 |
|  | ENRICHED IN T CELLS (I) (M7.0) | 60 | 2.15 | 0.0E+00 |
|  | MITOTIC CELL CYCLE IN STIMULATED CD4 T CELLS (M4.5) | 33 | 2.14 | 0.0E+00 |
|  | MITOCHONDRIAL CLUSTER (M235) | 17 | 2.13 | 0.0E+00 |
|  | RESPIRATORY ELECTRON TRANSPORT CHAIN (MITOCHONDRION) (M219) | 18 | 2.11 | 4.3E-05 |
|  | TBA (M174) | 22 | 2.05 | 4.0E-05 |
|  | ENRICHED IN B CELLS (I) (M47.0) | 48 | 2.03 | 3.7E-05 |
|  | E2F1 TARGETS (Q3) (M10.0) | 32 | 2.01 | 3.5E-05 |
|  | CELL DIVISION - E2F TRANSCRIPTION NETWORK (M4.8) | 19 | 2.01 | 3.3E-05 |
|  | DNA REPAIR (M76) | 22 | 2.00 | 3.1E-05 |
|  | MITOTIC CELL CYCLE (M4.7) | 21 | 2.00 | 6.0E-05 |
|  | CELL DIVISION IN STIMULATED CD4 T CELLS (M4.6) | 19 | 1.99 | 5.7E-05 |
|  | E2F1 TARGETS (Q4) (M10.1) | 21 | 1.93 | 4.0E-04 |
|  | T CELL SURFACE SIGNATURE (S0) | 25 | 1.90 | 7.5E-04 |
|  | T CELL DIFFERENTIATION (TH2) (M19) | 16 | 1.90 | 7.4E-04 |
|  | PLASMA CELLS, IMMUNOGLOBULINS (M156.1) | 15 | 1.88 | 8.5E-04 |
|  | ENRICHED IN B CELLS (II) (M47.1) | 36 | 1.88 | 8.4E-04 |
|  | MITOTIC CELL CYCLE IN STIMULATED CD4 T CELLS (M4.9) | 15 | 1.85 | 1.6E-03 |
|  | PLASMA CELLS & B CELLS, IMMUNOGLOBULINS (M156.0) | 25 | 1.84 | 1.7E-03 |
|  | T CELL ACTIVATION (II) (M7.3) | 30 | 1.84 | 1.7E-03 |
|  | TBA (M153) | 15 | 1.75 | 4.8E-03 |
|  | ENRICHED IN B CELLS (VI) (M69) | 20 | 1.74 | 5.4E-03 |
|  | CELL DIVISION (STIMULATED CD4+ T CELLS) (M46) | 28 | 1.70 | 8.3E-03 |
|  | CELL CYCLE, ATP BINDING (M144) | 16 | 1.70 | 8.2E-03 |
|  | ENRICHED IN NK CELLS (I) (M7.2) | 46 | 1.68 | 9.7E-03 |
|  |  |  |  |  |
| Negative | IMMUNE ACTIVATION - GENERIC CLUSTER (M37.0) | 322 |  | 1.0E+00 |
|  | ENRICHED IN MONOCYTES (II) (M11.0) | 185 | -4.57 | 0.0E+00 |
|  | ENRICHED IN NEUTROPHILS (I) (M37.1) | 47 | -4.31 | 0.0E+00 |
|  | TLR AND INFLAMMATORY SIGNALING (M16) | 43 | -4.22 | 0.0E+00 |
|  | MONOCYTE SURFACE SIGNATURE (S4) | 87 | -3.95 | 0.0E+00 |
|  | ENRICHED IN MONOCYTES (IV) (M118.0) | 53 | -3.56 | 0.0E+00 |
|  | ENRICHED IN ACTIVATED DENDRITIC CELLS (II) (M165) | 35 | -3.15 | 0.0E+00 |
|  | BLOOD COAGULATION (M11.1) | 22 | -3.06 | 0.0E+00 |
|  | ENRICHED IN ACTIVATED DENDRITIC CELLS/MONOCYTES (M64) | 16 | -3.03 | 0.0E+00 |
|  | ACTIVATED (LPS) DENDRITIC CELL SURFACE SIGNATURE (S11) | 37 | -2.86 | 0.0E+00 |
|  | MYELOID CELL ENRICHED RECEPTORS AND TRANSPORTERS (M4.3) | 31 | -2.86 | 0.0E+00 |
|  | ENRICHED IN MONOCYTES (SURFACE) (M118.1) | 15 | -2.61 | 0.0E+00 |
|  | ANTIVIRAL IFN SIGNATURE (M75) | 22 | -2.53 | 0.0E+00 |
|  | REGULATION OF SIGNAL TRANSDUCTION (M3) | 47 | -2.47 | 0.0E+00 |
|  | TBA (M105) | 18 | -2.39 | 2.9E-04 |
|  | ENDOPLASMIC RETICULUM (M37.2) | 19 | -2.34 | 6.1E-04 |
|  | COMPLEMENT ACTIVATION (I) (M112.0) | 17 | -2.32 | 7.4E-04 |
|  | TRANSMEMBRANE TRANSPORT (I) (M87) | 24 | -2.27 | 9.1E-04 |
|  | VIRAL SENSING & IMMUNITY; IRF2 TARGETS NETWORK (I) (M111.0) | 17 | -2.24 | 9.7E-04 |
|  | EXTRACELLULAR MATRIX (II) (M2.1) | 45 | -2.19 | 1.6E-03 |
|  | TBA (M114.0) | 37 | -2.17 | 1.7E-03 |
|  | REGULATION OF ANTIGEN PRESENTATION AND IMMUNE RESPONSE (M5.0) | 81 | -2.16 | 1.7E-03 |
|  | SMALL GTPASE MEDIATED SIGNAL TRANSDUCTION (M215) | 16 | -2.10 | 3.3E-03 |
|  | ENRICHED IN MYELOID CELLS AND MONOCYTES (M81) | 35 | -2.09 | 3.2E-03 |
|  | CHEMOKINES AND INFLAMMATORY MOLECULES IN MYELOID CELLS (M86.0) | 18 | -2.07 | 3.7E-03 |
|  | INTERFERON ALPHA RESPONSE (I) (M158.0) | 16 | -1.95 | 6.9E-03 |

From the database genes were ranked based on their correlation with PEBP1 expression and used as input for GSEA using blood transcriptional modules (BTMs) as gene sets. NES: normalized enrichment factor FDR: false discovery rate. Shown are gene sets enriched in genes positively (upper part) or negatively (lower part) correlating with PEBP1 expression.
